# Ameboma: an unusual cause of gastrointestinal bleeding during severe leptospirosis

**DOI:** 10.1186/1471-2334-14-299

**Published:** 2014-06-02

**Authors:** Tristan Legris, Marie-Christine Jaffar-Bandjee, Olivier Favre, Nicole Lefrançois, Robert Genin, Claire Ragot, Carla Fernandez, Anne-Hélène Reboux

**Affiliations:** 1 Service de Néphrologie, Dialyse et Transplantation Rénale, Centre Hospitalier Universitaire Felix Guyon, Saint-Denis, La Réunion, France; 2 Service de Microbiologie, Centre Hospitalier Universitaire Felix Guyon, Saint-Denis, La Réunion, France; 3 Service d’Hépato-Gastro-Entérologie, Centre Hospitalier Universitaire Felix Guyon, Saint-Denis, La Réunion, France; 4 Service de Réanimation Polyvalente, Centre Hospitalier Universitaire Felix Guyon, Saint-Denis, La Réunion, France; 5 Service de Pathologie, Centre Hospitalier Universitaire Felix Guyon, Saint-Denis, La Réunion, France; 6 Assistance Publique Hôpitaux de Marseille, Hôpital Conception, Centre de Néphrologie et Transplantation Rénale, 147 Bd Baille, 13385, Marseille Cedex 05, France

**Keywords:** Leptospirosis, Weil’s disease acute renal failure, Amebiasis, Coinfection

## Abstract

**Background:**

Severe leptospirosis occurs mainly in a tropical environment and includes icterus, acute renal failure and hemorrhages. These bleedings, which are mainly a consequence of acute homeostatic disturbances, can also reveal simultaneous diseases. Coinfections with other tropical diseases have been previously reported during leptospirosis. To our knowledge, invasive amebiasis, which can induce gastrointestinal bleedings, has never been described in the course of severe leptospirosis.

**Case presentation:**

In this report, we describe a case of a 60 year-old man living in Reunion Island (Indian Ocean, France) admitted to our intensive care unit for severe *Leptospira interrogans* serovar *icterohaemorrhagiae* infection with neurological, renal, liver and hematological involvement. Two lower gastrointestinal bleedings occurred 7 and 15 days after admission. The first episode was promoted by hemostatic disturbances while the second bleeding occurred during low-dose heparin therapy. Colonoscopy revealed a pseudo-tumoral inflammatory mass of the recto-sigmoid junction. Histological examination found trophozoites inside mucinous exudate suggestive of *Entamoeba histolytica*. Amoebic serology was strongly positive whereas careful detection of cysts or trophozoites on saline-wet mount was negative in three consecutive samples of stools. Amoxicillin followed by metronidazole therapy, combined with supportive care, led to an improvement in the clinical and biological patient’s condition and endoscopic appearances.

**Conclusion:**

Clinicians should be aware that gastrointestinal bleeding during severe leptospirosis could not solely be the consequences of hemostatic disturbances. Careful endoscopic evaluation that may reveal curable coinfections should also be considered.

## Background

Leptospirosis is a worldwide zoonosis caused by pathogenic *Leptospira* species which occurs predominantly in warm and humid climates [1]. Direct or indirect contact with urine of infected rodents or other animals can induce human leptospirosis whose clinical spectrum can substantially vary from one patient to another [2]. Most infections are mild, characterised by sudden fever (97%), chills (78%), headache (98%), myalgia with rhabdomyolysis (79%), arthralgia (78%), gastrointestinal symptoms (35%), cough (20%) and rash (7%) that can mimic many other infections [3]. Aseptic meningitis has also been reported in up to one-quarter of cases [4]. Weil’s disease, including jaundice, acute renal failure and hemorrhage represents the most severe form of the illness [5], with mortality rates ranging from 5-15%. Pulmonary hemorrhage (occasionally complicated by adult respiratory distress syndrome), myocarditis and autoimmune-associated anterior uveitis [6] are also classical clinical features reported in severe leptospirosis cases.

Leptospirosis can also induce hemostasis disorders [7] such as thrombocytopenia (reported in 50-80% of patients), disseminated intravascular coagulation and more scarcely reported, haemolytic uraemic syndrome and thrombotic thrombocytopenic purpura. Endothelial dysfunction seems to play a crucial role in the loss of hemostasis during severe leptospirosis [7]. Even if their pathophysiology is not fully understood, hemorrhagic complications are reported to affect 20-60% of patients suffering from severe leptospirosis, with an incidence of less than 10% of gastrointestinal bleeding [8,9]. One recent prospective study found a high incidence (46%) of mild skin and mucosal bleeding whereas, urinary or gastrointestinal tract bleeding affected 13% of patients. A positive association between thrombocytopenia, increased prothrombin time and severe bleeding was also reported. Disseminated intravascular coagulation (DIC), which was not *per se* associated with bleeding or mortality, was found in 22% of patients [9].

Bleeding may affect leptospirosis prognosis especially during intra-alveolar events [10]. In our experience from Reunion Island (Indian Ocean, France), gastrointestinal hemorrhages were also reported to be a common cause of death during severe leptospirosis, especially in cases of concomitant acute renal failure [4].

We report here the case of a severe lower intestinal bleeding revealing ameboma during the course of Weil’s disease.

## Case presentation

**Figure 1:**
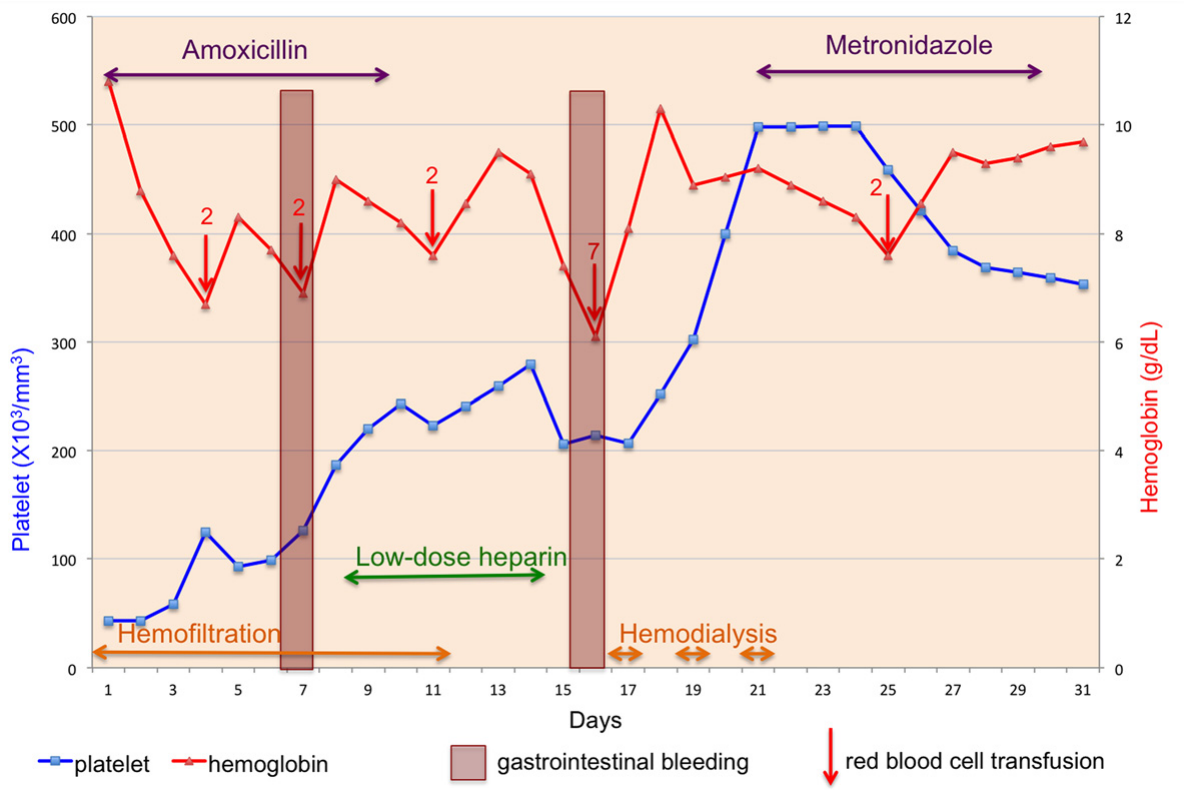
**Evolution of clinical and biological patient’s condition.** Platelet counts (blue line) and hemoglobin level (red line) during course of leptospirosis and amebiasis together with clinical gastrointestinal bleeding periods, red blood cell transfusions, antibiotic and renal replacement therapies. Red numbers above the arrows refer to the numbers of Units of packed cells transfused.

A 60 year-old male smoker was admitted in our teaching hospital for fever, intense asthenia, diffuse pain, myalgia, headache, oliguria and jaundice of one week’s duration. This patient was living in a rural area of Reunion Island and was working daily barefoot in sugar cane fields. At physical examination, cutaneous icterus and acute confusion (without neurological focal signs nor clinical evidence of meningitis) were noted. No hemorrhagic signs were reported at admission. Biological tests revealed severe acute renal failure (serum creatinine level = 1022 μmol/L) with hyponatremia at 126 mmol/l, thrombocytopenia (43000/mm^3^), leucocytosis (19600/mm^3^), mild cytolysis (alanine aminotransferase = 213 IU/L) and severe jaundice (serum bilirubin > 500 μmol/L). Slight rhabdomyolysis was also noticed (creatin phosphokinase = 4475 IU/l). Prothrombin ratio (PT) was in the normal range (70%) whereas activated partial thromboplastin time (APTT) was prolonged (59 s, normal range: 30–41 s). Fibrinogen level was high (8.9 g/l, normal range: 2–4 g/l). Lactate dehydrogenase and haptoglobin blood levels were normal; no schistocytes were seen on peripheral blood film. Cerebrospinal fluid (CSF) analysis showed evidence of meningoencephalitis (white blood cells = 20/mm^3^, protein level = 2.5 g/L). Chest radiography was normal. Ultrasonography of liver and kidneys was unremarkable. Three hemocultures performed during fever peaks were negative. Severe leptospirosis with muscular, neurological, liver, renal and hematological involvement was suspected and quickly confirmed by positive blood and urine PCR. Leptospirosis serology (ELISA) was positive, concomitantly with IgM against *Leptospira interrogans* serovar *icterohaemorrhagiae* strain Verdun (titer = 1/800). Leptospirosis polymerase chain reaction (PCR) in CSF was negative. Hepatitis A, B, C and E, human immunodeficiency virus (HIV) and dengue serologies were negative whereas chikungunya serology was positive for IgG without IgM indicating a past infection (blood chikungunya PCR was negative). *Rickettsia conorii*, *rickettsia typhi* and *coxiella burnetii* serologies were also negative.Given the initial severity, the patient was monitored in the Intensive Care Unit where neurological worsening lead to tracheal intubation and mechanical ventilation for 7 days. Parenteral amoxicillin (1 g t.i.d.) was administered for 10 days. Acute renal failure was treated with continuous veno-venous hemofiltration during 11 days, followed by 3 additional conventional hemodialysis sessions (Figure 1). Of note, a first episode of intestinal bleeding requiring the first red blood cell transfusion was reported on day 7. This bleeding complication unfortunately was unexplored. Because of reduced mobility, a persistent inflammatory state and the presence of a central dialysis catheter in the right femoral vein, low-dose prophylactic subcutaneous unfractionated heparin sodium (2500 units bid) was used between day 9 and day 14. Leptospirosis was considered to be effectively treated: recovery of renal function occurred after 21 days in hospital with a serum creatinine level slowly and constantly decreased to 329 μmol/l upon discharge. A slow improvement of serum liver profile was also observed (serum bilirubin at discharge = 44 μmol/L).

**Figure 2:**
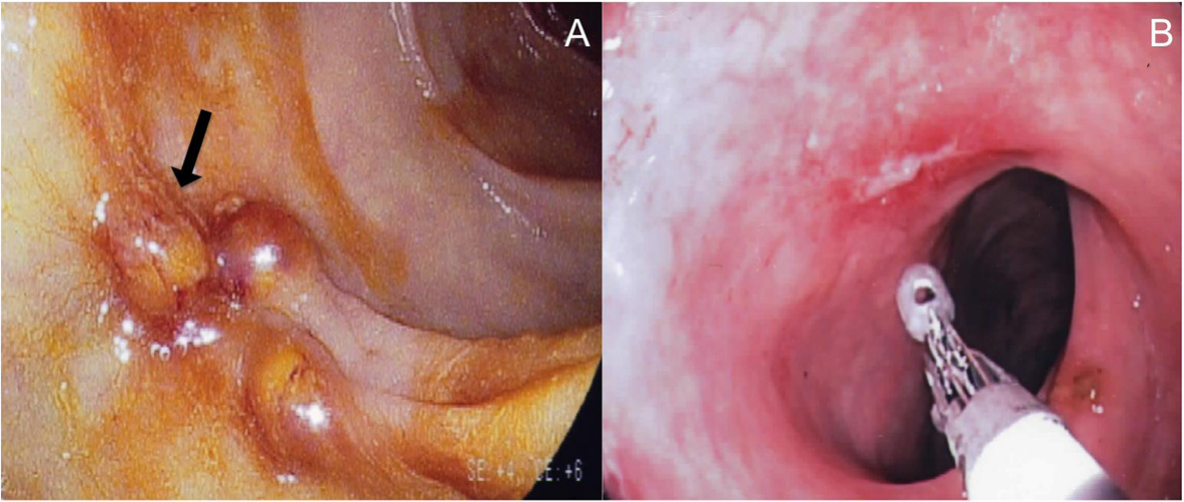
**Endoscopic findings. A**: First colonoscopy showing rectitis and a pseudo-tumoral nodules of recto-sigmoid junction (black arrow). **B**: Second colonoscopy, one month after the effective treatment of invasive amebiasis, revealed a small and healing ulceration of the middle rectum.

**Figure 3:**
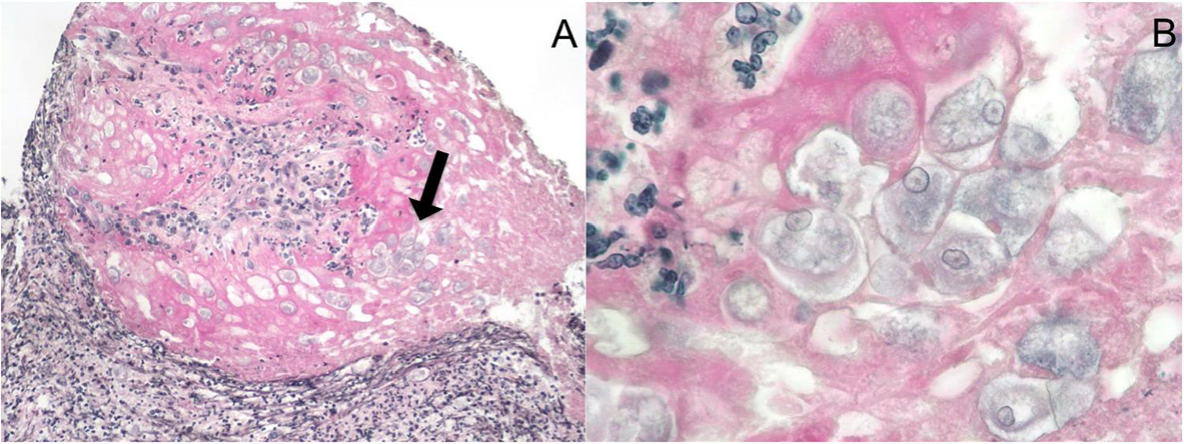
**Histological examination of rectal biopsy.** The ulcerative lesion with nonspecific mucosal thickening together with blue oval structures (black arrow) with eccentric nuclei was suggestive of *Entamoeba histolytica* trophozoites. **A**: hematoxylin-eosin, × 20. **B**: hematoxylin-eosin, × 40.

At day 15 (Figure 1), while thrombocytopenia had been completely resolved (259000/mm^3^), a second large lower gastrointestinal bleed with acute anemia was noticed (hemoglobin = 7 g/dL) requiring a red blood cell transfusion of 7 units. Standard hemostasis tests were normal. Upper gastrointestinal endoscopy was normal whereas colonoscopy revealed rectitis with pseudo-tumoral appearance of the recto-sigmoid junction (Figure 2A). Pathological examination of rectal biopsies found no neoplastic change, but rather a nonspecific mucosal thickening, acute ulceration together with blue oval structures inside mucinous exudate containing eccentric nuclei, suggesting the presence of *Entamoeba histolytica* trophozoites (Figure 3). Attempted careful detection of cysts or trophozoites on saline-wet mount was negative in three consecutive samples of stools. However, indirect hemagglutination amebiasis serology was strongly positive (titer = 1/2048) suggestive of invasive pseudo-tumoral colitis i.e. an ameboma. Oral metronidazole (500 t.i.d) was introduced for 10 days followed by luminal decontamination with tibroquinol-tiliquinol association (Intetrix®, the only luminal amebicide available in France). Repeated liver ultrasonography did not find evidence of an amebic abscess. Of note, blood cytomegalovirus (CMV) PCR was found positive (3.03 log) with positive IgG serology, without IgM. CMV immunostaining of colic biopsies did not reveal evidence of viral colitis. This mild blood CMV reactivation was considered as an asymptomatic reactivation and no antiviral therapy was started.One month after discharge, a repeat endoscopy revealed slight and healing ulceration of the middle rectum (Figure 2B). No new gastrointestinal bleeding was reported. The serum creatinine level was still decreasing to 156 μmol/L. Three months after discharge, the patient’s overall condition significantly improved and he felt able to work again. Physical examination was unremarkable. Serum creatinine was 111 μmol/L and amoebic serology titer was also decreasing (1/640). Serum liver enzymes were normal. Blood PCR for leptospirosis and CMV became negative.

## Discussion

Leptospirosis is a widespread zoonosis of global distribution but is more common in tropical areas where conditions for its transmission are particularly favourable. Although considered as an occupational disease, a higher proportion of cases related to recreational water based activity have been reported [3]. A mean of 55 annual confirmed cases were reported between 1998 and 2008 in Reunion Island with a high rate of infections between February and May, positively correlated with total rainfall, average temperature and global solar radiation [11]. *Leptospira* are highly motile aerobic spirochetes that can be directly transmitted via contact with infected tissues or contaminated urine, or indirectly by contact with water or soil contaminated with the urine of mammalian reservoirs. As is likely in our report, many infections can result from walking barefoot in damp conditions or gardening with bare hands [12]. Differential diagnosis of leptospirosis in Reunion Island should include chikungunya disease, whose incidence during the 2005–2006 outbreak was almost 40% [13], and dengue fever, whose overall seroprevalence in a recent blood donors survey was 3.5% [14]. Well-known independent risk factors for severe forms of leptospirosis (such as current cigarette smoking, delay >2 days between the onset of symptoms and the initiation of antibiotherapy or *Leptospira interrogans* serogroup *icterohaemorrhagiae* as the infecting strain [15]) were all found in our reported case.

In severe forms, leptospirosis can be complicated by hemorrhages (typically ranging from minor conjunctival suffusions to more serious pulmonary involvement with hemoptysis) whose pathogenesis remains not fully elucidated. A review focusing on this issue concluded that the bleeding tendency results from an imbalance in the hemostatic equilibrium that may lead to DIC [7], which concerned nearly half of confirmed cases in Thailand [8]. In our report, initial laboratory tests allowed us to rule out DIC; PT was normal and only APTT was increased, a classical finding not reported to be associated with bleeding [8]. However, thrombocytopenia < 100000/mm^3^ at admission could have triggered the first intestinal bleed that unfortunately was not investigated by endoscopies. In recent papers, prevalence of thrombocytopenia < 100000/mm^3^ during the initial phase of leptospirosis was significantly higher among patients with than among those without bleeding [8,9]. Mechanisms of thrombocytopenia during leptospirosis are not fully understood: direct bone marrow toxicity of organism [16], platelet consumption due to DIC, immune-mediated causes [17] and platelet-activating aggregates related to direct endothelial damage of *Leptospira* (reviewed in [10]) were proposed. Thrombotic microangiopathy has been rarely reported during leptospirosis [18,19]. Unfortunately, bone marrow aspiration was not realized during the initial course of our patient’s history but peripheral thrombotic microangiopathy was ruled out (no evidence of hemolysis and no blood schistocytes upon admission). Finally, acute renal failure, which has been independently associated with mortality during leptospirosis [20], could have also contributed to these hemorrhages. Intrinsic platelet abnormalities, altered platelet-vessel wall interaction, dialysis and need of anticoagulant therapies are suggested factors that could promote bleeding diathesis during advanced kidney diseases [21]. In our report, we hypothesize that the second gastrointestinal bleed, occurring when platelet count was back to normal, could have possibly been triggered by a degree of prolonged uremic platelet dysfunction and by low-dose unfractionated heparin (Figure 1). Within the context of a recent unexplained gastrointestinal bleed and an acute infection known to predispose to haemorrhage, the balance between bleeding and thrombosis risk should be carefully weighted before introducing prophylactic anticoagulant therapy in critically ill patients [22].

During leptospirosis, coinfections with other tropical diseases (dengue fever, HIV, hepatitis A and E, scrub typhus, melioidosis and malaria [23-29]) have been previously reported but to our knowledge, our case represents the first simultaneous association of leptospirosis and amebiasis. *Entamoeba histolytica* is a protozoan parasite whose ingestion of cysts from contaminated food can induce amebiasis. Intestinal amebiasis has a worldwide distribution but disease is more frequently found in developing countries where risk of fecal contamination is high. In Reunion Island, only sporadic cases of amebiasis have been described [30]. During 2012 and 2013, on 60 serological tests performed in our laboratory, only 5 were positive for antiamebic antibodies (data non published). Self-limiting asymptomatic infections constitute 90% of cases whereas invasive colitis and amebic liver abscess represent only 10% and <1% of cases respectively. Invasive colitis results from excystation in the intestinal lumen producing trophozoites that can penetrate the mucous layer and epithelial cells [31]. It is well known that patients with invasive amebiasis may rarely develop tumorous, exophytic, solitary and inflammatory masses called “amebomas” that can be up to 15 cm in diameter. In cases of amebomas, stool examinations even if repeated are often negative whereas serological sensitivity is approximatively 70% positive [32]. It has been suggested that amebomas can occur in patients with long-standing, untreated or inadequately treated amebiasis [33]. In our case, we hypothesize that asymptomatic ameboma was probably present several weeks or months before admission and that leptospirosis-induced hemostasis disturbances allowed it to become symptomatic. Chronic clinical presentations include alternating diarrhea and constipation, weight loss, low-grade fever, cramping lower abdominal pain, palpable mass and obstructions. It can be endoscopically indistinguishable from inflammatory bowel disease, diverticulitis, pseudomembranous colitis or malignancy, especially in cases of concomitant liver abscess that might mimic metastatic colon cancer [34]. In most cases, after adapted drug therapy of ameboma, repeated colonoscopies reveal no abnormality [34].

## Conclusion

In conclusion, our report emphasises several key points that could improve management of patients with severe leptospirosis. Firstly, it highlights the importance of hemostasis disturbances and the need of a careful daily screening of bleeding consequences, especially during anticoagulation periods. Secondly, our report illustrates that bleeding may not only be the consequence of coagulation disorders during leptospirosis, and should be rigorously explored as early as possible since coincident diseases can be easily diagnosed and cured. We would recommend that severe gastrointestinal bleeding (defined by the need of transfusion support or hemodynamic instability together with acute anaemia) during leptospirosis should be promptly explored by oesophagogastroduodenoscopy and/or colposcopy as it is recommended in the general population. Thirdly, even if clinical features are sometimes unreliable to distinguish between them, two tropical infections can coexist in high-risk patients. As in our report, acute infections such as leptospirosis can unmask latent diseases such as amebomas.

## Consent

Written informed consent was obtained from the patient for publication of this Case report and its accompanying images. A copy of the written consent is available for review by the Editor of this journal.

## Abbreviations

APTT: Activated partial thromboplastin time; CMV: Cytomegalovirus; CSF: Cerebrospinal fluid; DIC: Disseminated intravascular coagulation; ELISA: Enzyme-linked immunosorbent assay; HIV: Human immunodeficiency virus; PCR: Polymerase chain reaction; PT: Prothrombin time.

## Competing interests

This work was supported by a grant from IP Santé, Marseille, France.

## Authors’ contributions

TL cared for the patient, collected data, carried out the literature search and drafted the manuscript. MCJB did the laboratory work and made critical revisions of the manuscript. OF performed intestinal endoscopies and made critical revisions of the manuscript. NL, CR and RG took care of the patient and made critical revisions of the manuscript. CF performed histological examinations and made critical revisions of the manuscript. AHR cared for the patient, collected data and made critical revisions of the manuscript. All authors read and approved the final manuscript.

## Pre-publication history

The pre-publication history for this paper can be accessed here:

http://www.biomedcentral.com/1471-2334/14/299/prepub
